# Worthless Quinia Pills

**Published:** 1875-02

**Authors:** 


Worthless Quinia Pills.—At a late meeting of the Pharmaceutical Society of this city, Dr. Miller cautioned against the purchase of cheap sugar-coated quinia pills. There were in the market 45,000 such pills which do not contain a trace of quinia. They were made from muriate of cinchonia, furnished by a New York house as sulphate of quinia to some of the makers of sugar-coated pills, and by them thrown back on the hands of the dealer, upon the discovery of the fraudulent nature of the article.—*Medical and Surgical Reporter.*

